# Effect of PEG Molecular Weight on the Polyurethane-Based Quasi-Solid-State Electrolyte for Dye-Sensitized Solar Cells

**DOI:** 10.3390/polym14173603

**Published:** 2022-09-01

**Authors:** Kai Sing Liow, Coswald Stephen Sipaut, Rachel Fran Mansa, Mee Ching Ung, Shamsi Ebrahimi

**Affiliations:** Faculty of Engineering, Universiti Malaysia Sabah, UMS Road, Kota Kinabalu 88400, Malaysia

**Keywords:** electrolyte, nanosilica, polyurethane, polyaniline, dye-sensitized solar cells

## Abstract

Nanosilica was surface modified with polyaniline and incorporated into polyurethane to form a polymer matrix capable of entrapping a liquid electrolyte and functioning as quasi-solid-state electrolyte in the dye-sensitized solar cells. The effect on the S−PANi distribution, surface morphology, thermal stability, gel content, and structural change after varying the PEG molecular weight of the polyurethane matrix was analyzed. Quasi-solid-state electrolytes were prepared by immersing the polyurethane matrix into a liquid electrolyte and the polymer matrix absorbency, conductivity, and ion diffusion were investigated. The formulated quasi-solid-state electrolytes were applied in dye-sensitized solar cells and their charge recombination, photovoltaic performance, and lifespan were measured. The quasi-solid-state electrolyte with a PEG molecular weight of 2000 gmol^−1^ (PU−PEG 2000) demonstrated the highest light-to-energy conversion efficiency, namely, 3.41%, with an open-circuit voltage of 720 mV, a short-circuit current of 4.52 mA cm^−2^, and a fill factor of 0.63.

## 1. Introduction

Dye-sensitized solar cells (DSSCs) are a promising renewable energy source due to their low manufacturing cost, ease of fabrication, and high efficiency [1]. Conventional DSSCs are mostly based on liquid electrolytes and the lifespan is a challenging issue. Liquid electrolytes suffer from solvent evaporation and leakage, which eventually reduce the DSSCs lifespan [2,3,4]. Various studies have been carried out to solve these problems, for instance, by using solid or gel electrolytes, ionic liquids, and polymer matrix-based quasi-solid-state electrolytes [4,5,6]. The penetration of solid electrolytes, gel electrolytes, and ionic liquids into the dye-sensitized semiconductor layer and their ion diffusion are poor, resulting in a poor DSSC efficiency although the lifespan is significantly improved.

The use of polymer matrix-based quasi-solid-state electrolytes seems to have a great potential for solving the penetration and ion diffusion problems. This is because the liquid electrolyte (high ion diffusivity and penetrability) absorbed inside the polymer matrix penetrates the dye-sensitized semiconductor layer when squeezed during the DSSC assembly process. Numerous polymers have been studied as the polymer matrix for quasi-solid-state electrolytes in the DSSCs, such as polyacrylic acid [7,8,9,10,11,12,13,14,15], polyvinylidene fluoride [16,17,18,19,20], polyacrylonitrile [21,22,23,24], polyvinyl butyral [25], polyhydroxyethyl acrylate/polyethylene glycol [26], cellulose acetate [27], polycarbonate [28], polyurethane [29,30], and so on. Polyacrylic acid (PAA)- and polyvinylidene fluoride (PVDF)-based quasi-solid-state electrolytes are the most studied.

PAA is a superabsorbent. However, it is not a good absorbent when it is used as the liquid electrolyte of the DSSCs. This is because PAA consists of the −COOH group, whereas the common solvent for the liquid electrolyte (i.e., propylene carbonate, ethylene carbonate and gamma-butyrolactone, N-methyl-2-pyrrolidone and 3-methoxypropionitrile) consists of the –C=O, C–O–C or –CN group. To improve the liquid electrolyte absorbency, PAA was grafted with polyethylene glycol [7,8,9,10,11,12], cetyltrimethylammonium bromide [13,14], gelatin [15], and so on. The efficiency of the DSSCs based on PAA ranged from 1.61% to 9.10% [8,9,11,12,13,14,15,31]. 

PVDF attracts attention due to its high dielectric constant, good electrochemical stability in the presence of titanium dioxide and platinum, good affinity towards the liquid electrolyte because of the electron-withdrawing fluorine atoms in the polymer backbone, and it is conductive at room temperature (10^−4^ to 10^−3^ Scm^−1^) [18,19,32]. However, the crystalline property of PVDF hinders the movement of the ions, which reduces the conductivity of the formulated electrolyte [18,33]. Therefore, many studies have been conducted to reduce the crystallinity. For example, PVDF was copolymerized with hexafluoropropylene, namely, PVDF−HFP. The crystallinity of PVDF was around 45.8% to 54.8% [34,35,36], and after being copolymerized with hexafluoropropylene, was around 27.7% to 28.8% [34,37]. The incorporation of nanoparticles into PVDF−HFP was also widely conducted to reduce the crystallinity. A huge increase in DSSC efficiency from 3.09% to 7.75% was reported upon the incorporation of vanadium pentoxide [32], whereas the efficiency was from 5.02% to 5.96% upon the incorporation of titanium dioxide [20] and from 4.57% to 5.15% upon the incorporation of barium titanate [38]. 

Polyurethane (PU) attracts attention due to its ability to absorb liquid electrolytes. PU has a unique structure due to its two-phase morphology, i.e., soft and hard segments. The main components in PU are polyol and isocyanate compounds. The soft segment is contributed by polyol, whereas the hard segment is from an isocyanate compound. In PU electrolytes, the soft phase acts as a polymeric solvent to solvate the redox couple (I^−^/I_3_^−^) in the liquid electrolyte. It also has high segmental motion which facilitates the mobility of ions. The hard phase of the PU distributed or interconnected throughout the soft phase acts as a physical crosslinker and filler to the soft phase and contributes to the dimensional stability of the polymeric matrix [39]. Despite the great potential of PU, only a few studies were found that reported an efficiency ranging from 0.8% to 7.68% [29,30,39,40,41,42,43,44,45] and which mostly focused on the type of polyol and incorporation of nanoparticles.

There is no study reporting on the effect of the polyol molecular weight on the performance of the DSSCs. For the DSSCs based on the polymer matrix’s quasi-solid-state electrolyte, the absorbency of the polymer matrix towards the liquid electrolyte is a key factor that affects the DSSC performance. The polyol molecular weight of the PU matrix strongly affects the polymer matrix absorbency. Therefore, the novelty of this study is the investigation of the effect of the polyol (polyethylene glycol) molecular weight (10,000, 8000, 6000, 4000, and 2000 gmol^−1^) of PU on the performance of the formulated quasi-solid-state electrolyte (i.e., thermal stability, crystallinity, gel content, polymer matrix absorbency, conductivity, and ion diffusion coefficient) and the DSSC (i.e., charge recombination, efficiency, and lifespan).

## 2. Materials and Methods

Nitric acid, 69% (HNO_3_), sodium iodide (NaI), iodine (I_2_), propylene carbonate (PC), polyethylene glycol (PEG) with a molecular weight of 10,000, 8000, 6000, 4000, and 2000 gmol^−1^, 4,4′-diphenylmethane diisocyanate (MDI), 96% ethanol, and iso-propanol (IPA) were purchased from Merck. Titanium dioxide (TiO_2_, Aeroxide P25), 4-(1,1,3,3-Tetramethylbutyl)phenyl-polyethylene glycol (Triton X-100), Di-tetrabutylammonium cis-bis(isothiocyanato)bis(2,2′-bipyridyl-4,4′-dicarboxylato) ruthenium (II) (N719), chloroplatinic acid hexahydrate (H_2_PtCl_6_·6H_2_O), and a fluorine-doped tin oxide coated glass slide (FTO) were purchased from Sigma-Aldrich, Melbourne. 

### 2.1. Preparation of the Polyurethane Quasi-Solid-State Electrolyte (QSE)

To prepare the quasi-solid-state electrolyte, S−PANi was synthesized via the post-modification method [29]. The prepared S−PANi was sonicated in 1 mL of PC for 1 h. As shown in Table 1, 1 g of PEG was then added into the solution and stirred for 2 h at 65 °C. After that, 0.3 g MDI was added dropwise into the solution under continuous stirring [46]. The resulting solution was casted on a glass plate and cured at room temperature (23–25 °C) for 2 h [47]. The glass plate was masked with scotch tape to control the thickness of the polymer matrix at 100–110 µm. The polymer matrix was detached from the glass plate and dried in an oven at 60 °C for 24 h [11]. After drying, the polymer matrix was immersed into the LE (1.5 g NaI, 0.15 g I_2_, and 15 mL PC) for 2 days. The label and formulation of the formulated polymer matrices are shown in Table 1.

### 2.2. Preparation of Dye-Sensitized Solar Cells

The preparation of the DSSCs was divided into three parts, which included the photoanode, electrolyte, and counter electrode [8,12,19,48]. To prepare the photoanode: TiO_2_ paste was prepared by mixing 0.25 g of TiO_2_, 1.25 mL of HNO_3_ (0.1 M), 0.13 g of PEG 20,000, and 20 µL of Triton X for 15 min. After that, the TiO_2_ paste was coated onto the FTO glass and sintered at 450 °C for 60 min. Then, it was immersed in N719 solution (0.035 wt% of N719 in ethanol) for 24 h. To prepare the platinized counter electrode: 20 µL of H_2_PtCl_6_ (H_2_O)_6_ solution (0.5 wt% of H_2_PtCl_6_ (H_2_O)_6_ in IPA) was dropped onto the conductive side of the FTO glass and sintered at 450 °C for 45 min. To prepare the DSSCs: a slice of the formulated electrolyte (0.125 cm^2^) was sandwiched in between the prepared photoanode and the platinized counter electrode. A simulator was set up to measure the photovoltaic performance of the DSSCs. The simulator was equipped with a metal-halide lamp [49,50,51,52] (150 W) and the solar irradiation was set at 60 mWcm^−2^. The solar irradiation was calibrated with a power meter (TES, TES-1333R). The photovoltaic performance of the DSSCs was measured from 0 V to 1 V at a scan rate of 10 mVs^−1^.

### 2.3. Characterization

A microscope (Olympus BX60M) equipped with a camera (Paxcam) was used to investigate the surface morphology and porosity. The thermal property of the samples was studied by thermogravimetric analysis (PerkinElmer Pyris 6 TGA). The sample (5–10 mg) was heated under a nitrogen atmosphere condition at a heating rate of 10 °Cmin^−1^ from 30 °C to 600 °C [47,53,54,55,56]. The crystallinity of the dried sample was studied via a monochromatic, high-intensity X-ray diffractometer (XRD, SIEMENS D5000) that used Cu-Kα radiation (k = 1.54056 Å, 40 kV, 30 mA) at room temperature from a diffraction angle (2θ) of 5° to 80° (the increment step was 0.02° and the step time was 0.5 s) [54,55,57]. The liquid electrolyte absorbency of the polymer matrix was measured with a digital analytical balance and calculated with the following Equation (1) [9,20,48]:

(1) $$\mathrm{Liquid}\;\mathrm{Aborbency}\;\left({\mathrm{gg}}^{-1}\right)=\frac{\mathrm{Final}\;\mathrm{Weight}\;-\;\mathrm{Initial}\;\mathrm{Weight}}{\mathrm{Initial}\;\mathrm{Weight}}$$

where the initial weight is the weight of the polymer matrix before being immersed into the liquid electrolyte and the final weight is the weight of the polymer matrix after being immersed into the liquid electrolyte. To measure the conductivity [58,59,60], the QSE was sandwiched in between two platinized FTO glasses (Pt|QSE|Pt). The conductivity (σ) measurement was carried out by the AC impedance method using an LCR meter (GW INSTEK, LCR-821) with an applied voltage of 20 mV that ranged from 10 Hz to 100 kHz at 23–25 °C. σ was calculated by using Equation (2):

(2) $$\sigma =\frac{L}{A.R_{b}}$$

where L is the thickness of the QSE, A is the area of the sample electrode, and R_b_ is the bulk resistance. The ion diffusion coefficient (D_ff_) measurement was performed by the cyclic voltammetry method using the GSTAT101 (Autolab) with a scan rate of 50 mV that ranged from of −0.8 V to 0.8 V [61,62,63,64] at 23–25 °C. D_ff_ was calculated using the Randles–Sevcik equation as shown below:

(3) $$I_{p}=268600\times n^{\frac{3}{2}}\times v^{\frac{1}{2}}\times D_{\mathrm{ff}}{}^{\frac{1}{2}}\times A\times C$$

where I_p_ is the maximum current; n is the number of electrons transferred in the redox event; v is the scan rate; A is the electrode area; and C is the concentration of the electroactive species. The gel content test was conducted to measure the crosslinking degree of the sample. The sample was prepared according to the ASTM D2765-95. This method is used with standard procedures by refluxing the samples in boiling xylene for 24 h. The sample was placed in 100 mesh cages, labeled, and weighed. After refluxing in xylene, the residual solvent was removed at a temperature of 140 °C and placed for 4 h in an oven. The cages were removed, allowed to cool, and reweighed. The gel content was calculated as:

(4) $$\mathrm{Gel}\;\mathrm{Content}=\left(1-\frac{W_{3}-W_{e}}{W_{2}-{\;W}_{1}}\right)\times 100\%$$

where W_1_ is the weight of the mesh cage, W_2_ is the weight of the mesh cage and sample, W_3_ is the weight of the stapled cage and sample, and W_4_ is the weight of the stapled cage and sample after extraction and drying. The dark current measurement was conducted to measure the charge recombination of the DSSC [65,66,67,68]. It was measured under a dark condition from 0 V to 0.8 V at a scan rate of 10 mVs^−1^. The data were collected by the Autolab PGSTAT101 with NOVA software used to measure and calculate various performance parameters.

## 3. Results and Discussion

### 3.1. ATR−FTIR Analysis

**Figure 1:**
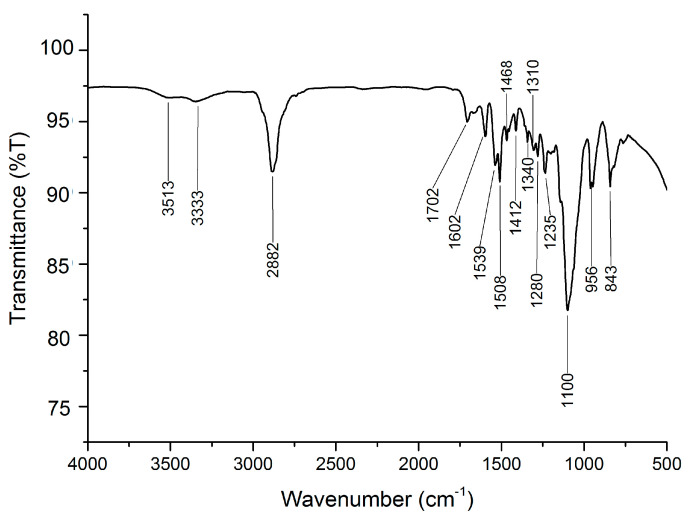
ATR−FTIR analysis of PU−PEG 10,000.

Figure 1 shows the ATR−FTIR spectra of PU−PEG 10,000 (only ATR−FTIR spectra of PU−PEG 10,000 are presented, as the spectra of PU−PEG 8000, PU−PEG 6000, PU−PEG 4000, and PU−PEG 2000 were similar). The ATR−FTIR shows the presence of –OH vibration (3513 cm^−1^), N–H vibrations (3333 and 1539 cm^−1^), –CH_2_ vibration (2882 cm^−1^), C=O vibration (1702 cm^−1^), C=C vibrations (1602, 1508, and 1468 cm^−1^), C–N vibration (1310 cm^−1^), C–H vibrations (1412, 1340, 1280, and 1235 cm^−1^), C–O–C and Si–O–Si vibration (1100 cm^−1^), and C–C vibrations (956 and 843 cm^−1^). This was in agreement with the chemical structure of PU. The occurrence of the N–H group, C=O group, and C–O–C group as well as the disappearance of the N=C=O group (2267 cm^−1^) [30] of MDI proved the formation of the PU matrix [69,70,71,72,73,74].

### 3.2. Distribution Pattern

**Figure 2:**
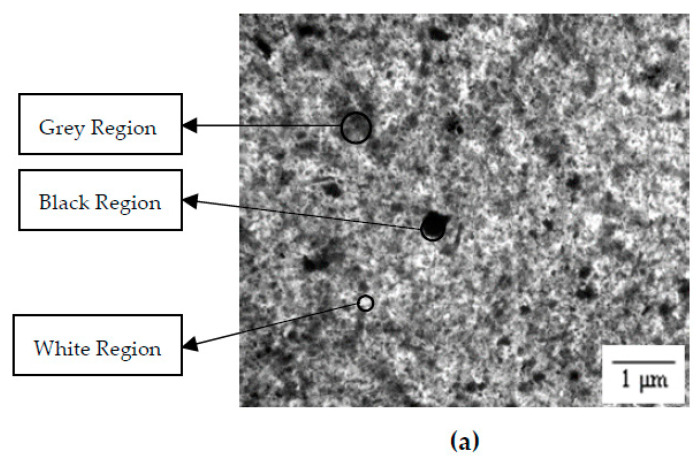

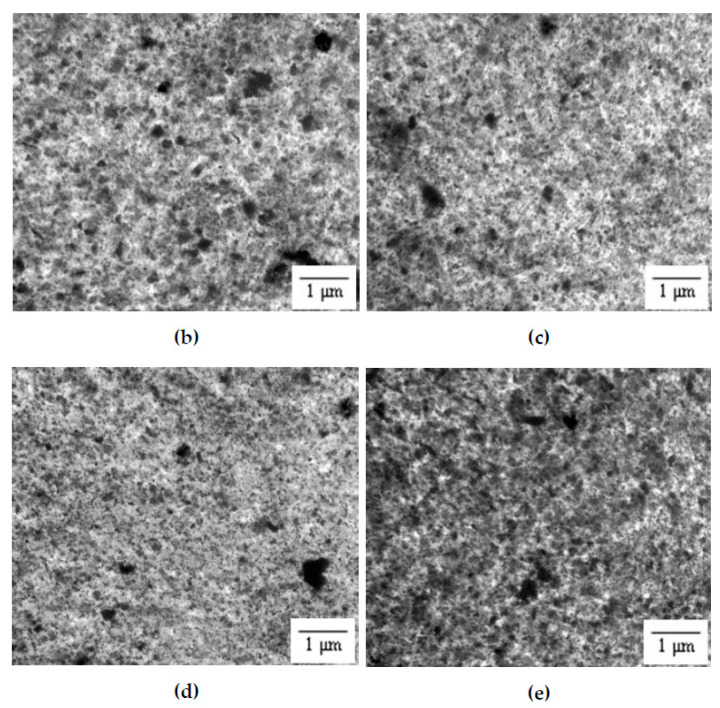
Transmitted light microscopic image of (**a**) PU−PEG 10,000, (**b**) PU−PEG 8000, (**c**) PU−PEG 6000, (**d**) PU−PEG 4000, and (**e**) PU−PEG 2000.

A transmitted light microscope was used to investigate the distribution pattern of S−PANi in the PU matrix of different PEG molecular weights. The images obtained are shown in Figure 2. It is highlighted that the black region indicates highly aggregated S−PANi, the grey region indicates evenly distributed S−PANi, and the white region indicates few or no S−PANi present in the PU matrix. The images show that the aggregated S−PANi became less and the distribution was more homogenous as the PEG molecular weight decreased. This is because the viscosity of the polymer solution decreased as the PEG molecular weight decreased [75,76,77]. In a less viscous medium, the aggregated particle breaks down further into smaller particles under mechanical force (stirring or sonication).

### 3.3. Surface Morphology

**Figure 3:**
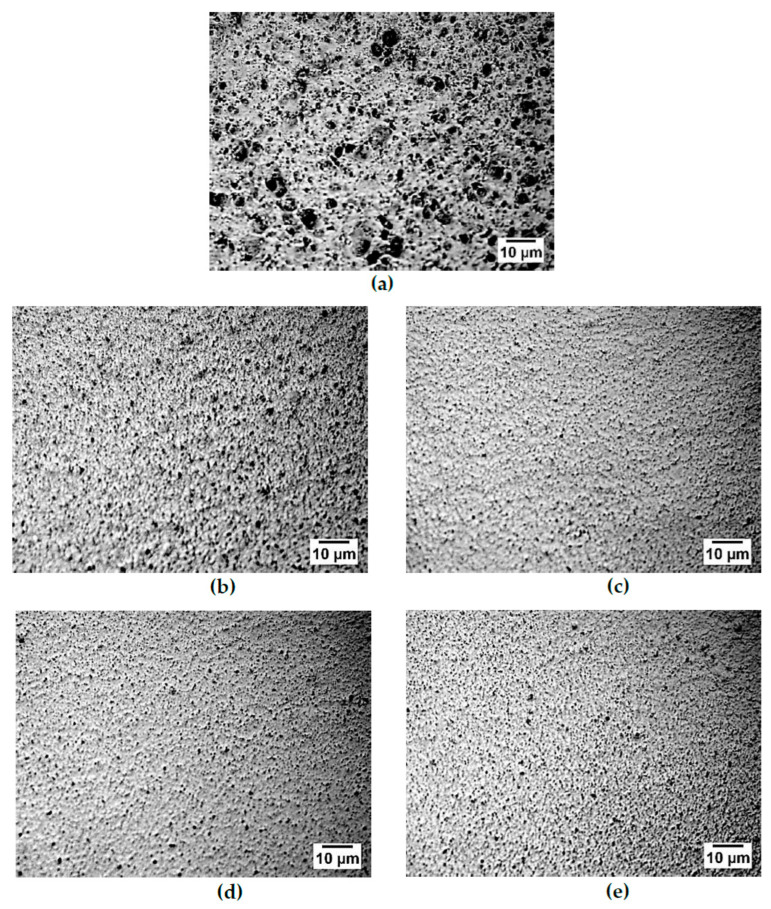
Reflected light microscopy image of (**a**) PU−PEG 10,000, (**b**) PU−PEG 8000, (**c**) PU−PEG 6000, (**d**) PU−PEG 4000, and (**e**) PU−PEG 2000.

A reflected light microscope was used to investigate the surface morphology of the PU matrix (Figure 3). Pores of different sizes were found on the surface of the PU matrices. The pore formation was due to phase separation which occurred due to the crosslinking of the S−PANi with the PU, as well as the incompatibility of the soft (PEG) and hard segment (MDI) in the PU [57,78,79]. It was also due to the hydrogen bonding-induced polymeric-rich and solvent-rich region in the PU matrices, which upon drying, caused the solvent in the solvent-rich region to evaporate [80]. As the molecular weight of the PEG increased, the pore sizes and the number of pores increased. Increasing the PEG molecular weight increased the soft and hard segment length, and promoted the incompatibility of the soft and hard segment [81,82,83] and crosslinking level, thereby increasing the pore formation.

### 3.4. Thermal Stability

**Figure 4:**
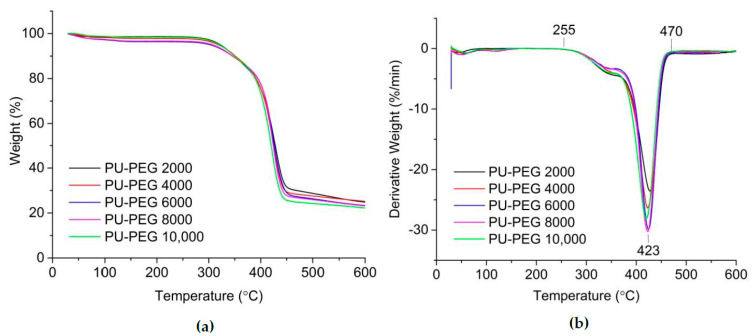
(**a**) TGA and (**b**) DTG thermograms of PU matrices of different PEG molecular weights.

Figure 4a and b show the TGA and DTG thermograms of the PU matrix with different PEG molecular weights. It can be seen that the thermal degradation trend for all of the PU matrices was similar. Varying the PEG molecular weight neither improved nor degraded the thermal stability of the PU matrix. All of the PU matrices were stable up to 255 °C, which were suitable to be applied in DSSCs for outdoor application. Two thermal degradation steps can be observed from the DTG thermograms. The thermal degradation steps were not separated from each other. The first thermal degradation was due to the breakage of the urethane bond (hard segment), whereas the second was due to the breakage of the ether bond (soft segment) in the PU matrix [53,54,84,85,86].

### 3.5. Structural Analysis

**Figure 5:**
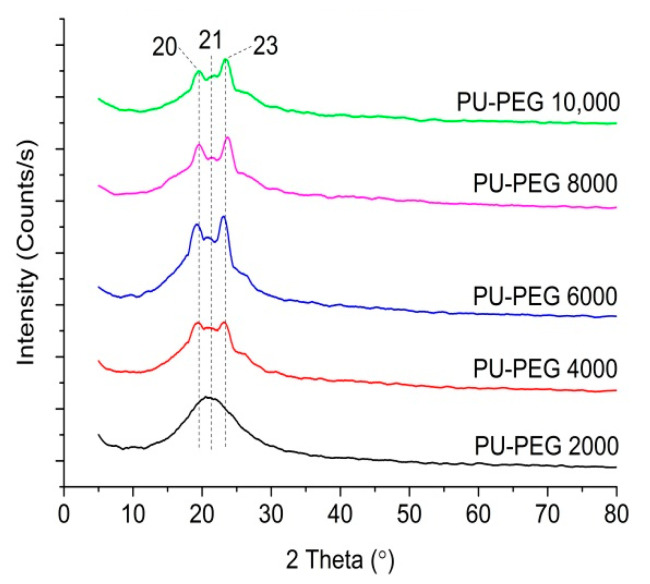
XRD spectra of PU matrices of different PEG molecular weights.

Figure 5 shows the XRD spectra of the PU matrix with different PEG molecular weights. All of the PU matrices show three diffraction peaks centered at 2θ = 20°, 21°, and 23°, indicating a semi-crystalline nature, except for in PU−PEG 2000. The XRD spectra of PU−PEG 2000 only show a broad diffraction peak centered at 2θ = 21°, indicating an amorphous nature. The degree of crystallinity of PU−PEG 10,000, PU−PEG 8000, PU−PEG 6000, PU−PEG 4000, and PU−PEG 2000 was 4.51%, 5.10%, 4.83%, 2.42%, and 0%, respectively. The degree of crystallinity was not significantly different from PU−PEG 10,000 to PU−PEG 6000, but significantly decreased from PU−PEG 4000 to PU−PEG 2000. PEG is a semi-crystalline material and the crystallinity increased with the molecular weight [87,88]. As the molecular weight of the PEG increased, the degree of crystallinity increased due to the low segmental mobility and a greater tendency to form an ordered alignment [89,90]. The increase in the crystallinity as the PEG molecular weight increased increases the crystallinity of the PU matrix. PU−PEG 2000 was amorphous despite PEG 2000 being semi-crystalline. This might be because there was a better dispersion of S−PANi in PU−PEG 2000, as mentioned earlier, preventing the polymer chain of PEG 2000 from being aligned orderly.

### 3.6. Gel Content

**Figure 6:**
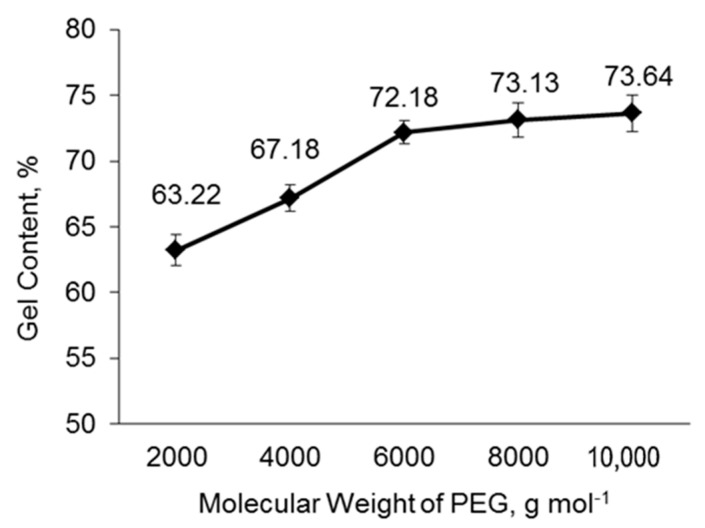
Gel content of PU matrices with different PEG molecular weights.

As shown in Figure 6, the average gel content increased significantly from PU−PEG 2000 to PU−PEG 6000, and then became less significant from PU−PEG 6000 to PU−PEG 10,000. The crosslinked networks are formed by the reaction between the −NH_2_/–NH groups of S−PANi and the –NCO groups of MDI [91,92,93,94]. Hence, as the PEG molecular weight increases, the –OH groups available to react with the –NCO groups decrease; therefore, more –NCO groups are available to react with the S−PANi, leading to more network formation. This is supported by the NCO:OH ratio of the sample, where the NCO:OH ratio for PU−PEG 10,000 is 12.0, PU−PEG 8000 is 9.6, PU−PEG 6000 is 7.2, PU−PEG 4000 is 4.8, and PU−PEG 2000 is 2.4. The insignificant increment of the crosslinking level as the PEG molecular weight increases from 6000 gmol^−1^ to 10,000 gmol^−1^ is due to the S−PANi becoming the limiting factor. An insufficient amount of S−PANi is available for the crosslinking reaction with the –NCO group.

### 3.7. Polymer Matrix Absorbency, Electrical Property, and Electrochemical Property

**Figure 7:**
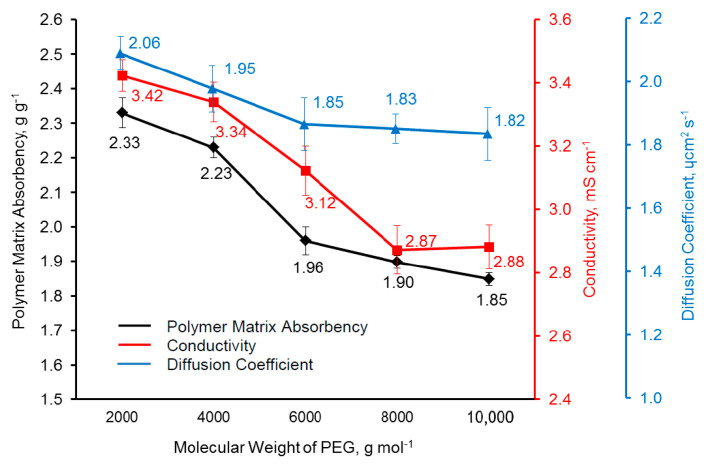
Polymer matrix absorbency, conductivity, and diffusion coefficient of PU matrices of different PEG molecular weights.

The polymer matrix absorbency of the PU matrix with different PEG molecular weights was measured (Figure 7). The polymer matrix absorbency decreased as the PEG molecular weight increased. This trend is in accordance with the trend of the gel content or crosslinking level. A high degree of crosslinking restricted the swelling ability of the PU matrix, thereby reducing the polymer matrix absorbency [95,96].

The conductivity of the PU matrix of different PEG molecular weights was measured, as shown in Figure 7. The conductivity decreased as the molecular weight of PEG increased from 2000 gmol^−1^ to 8000 gmol^−1^ and then became constant. The conductivity trend was similar to that of the polymer matrix absorbency trend. The conductivity of the PU matrix decreased as the amount of the LE uptake by the PU matrix decreased [97]. This is because fewer ions (Na^+^, I^−^ and I^−^_3_) and solvents were available to transfer the electron. This is also because S-PANi is more homogenously distributed as aforementioned, providing a more interconnected and continuous conductive path and leading to the increase in conductivity [98].

The ion diffusion coefficient of PU matrices with different PEG molecular weights is shown in Figure 7. The ion diffusion coefficient decreases as the PEG molecular weight increases from 2000 gmol^−1^ to 6000 gmol^−1^ and becomes constant as the PEG molecular weight increases from 6000 gmol^−1^ to 10,000 gmol^−1^. The ion diffusion coefficient correlated to the gel content, polymer matrix absorbency, and crystallinity level of the PU matrix. The trend of the ion diffusion coefficient is similar to that of the crosslinking level and polymer matrix absorbency trends. This is because ions are difficult to transfer through a highly crosslinked PU matrix [99,100]. As the polymer matrix absorbency decreases, there are fewer ions available to transfer; therefore, the ion diffusion coefficient decreases and vice versa. Besides that, as mentioned above, there was more aggregated S−PANi found in the PU matrix as the molecular weight of the PEG increased. The aggregated S−PANi hindered the diffusion of the ions. The ion diffusion coefficient also depends on the physical state of the polymer, i.e., whether it is amorphous or crystalline [69]. The ions are easy to diffuse in the amorphous region as the segmental movement of the polymer helps to transfer the ions. As aforementioned, the crystallinity of the PU matrix increased as the molecular weight of the PEG increased.

### 3.8. Charge Recombination of DSSCs

**Figure 8:**
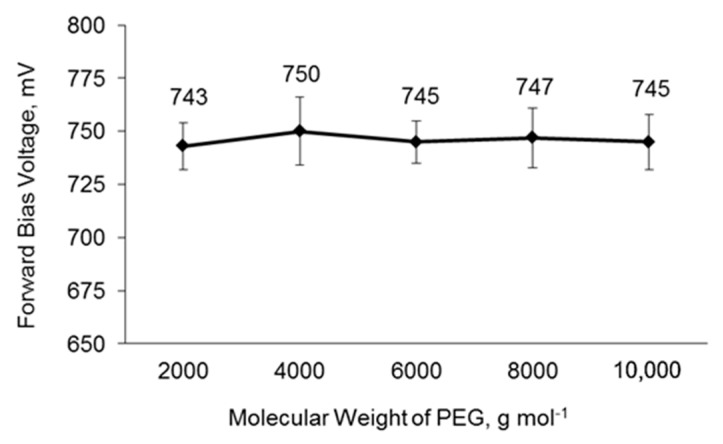
Forward bias voltage of DSSCs based on PU matrices with different PEG molecular weights.

Dark current analysis was conducted to investigate the charge recombination of the DSSCs (Figure 8). There is no change in the forward bias voltage as the PEG molecular weight increases. A high forward bias voltage indicates a low charge recombination and vice versa [101,102]. The charge recombination is correlated to the amount of the triiodide ion, whereas the amount of the triiodide ion is correlated to the polymer matrix absorbency. The decrease in the liquid electrolyte absorbency as the PEG molecular increases indicates a decrease in triiodide ion in the PU matrix, and suggests a decrease in charge recombination. This is because there is less triiodide ion to receive the electron back transfer from TiO_2_. It was found that the trend of charge recombination is not in accordance to the trend suggested; this is because the charge recombination is also affected by the ion diffusion coefficient. The decrease in the ion diffusion coefficient as the PEG molecular weight increases suggests an increase in the charge recombination. A low ion diffusion coefficient indicates slow ion movement in the PU matrix, which increases the chance of triiodide ions to receive the electron at the interface of the electrolyte and TiO_2_ layer. These factors contributed to the insignificant changes in the charge recombination as the PEG molecular weight varied.

### 3.9. Photovoltaic Performance of DSSCs

**Figure 9:**
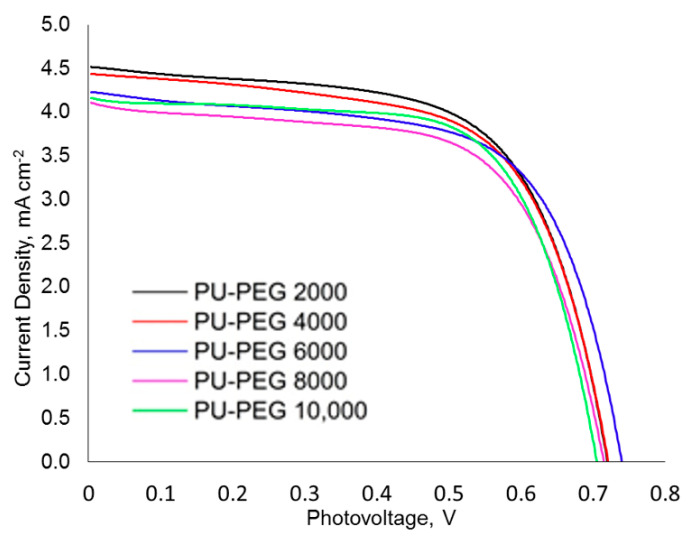
Current density-photovoltage curves of DSSCs based on PU matrices with different PEG molecular weights.

Figure 9 shows the current density vs. the photovoltage curve (IV curve) of the DSSCs based on the PU matrices with different PEG molecular weights. The V_oc_ and J_sc_ were extracted from the IV curves, whereas the FF and the efficiency of the DSSCs were calculated and presented in Table 2. The V_oc_ of DSSCs did not have any significant changes as the PEG molecular weight varied, as it was affected by the charge recombination. A low degree of charge recombination results in a higher V_oc_ and vice versa [103,104]. As the PEG molecular weight increases, the J_sc_ decreases up to 8000 gmol^−1^ and then remains stable as the PEG molecular weight increases further. The decrease in the J_sc_ is attributed to the decrease in the conductivity as the PEG molecular weight increases.

The J_sc_ decreases as the PEG molecular weight increases up to 8000 gmol^−1^ and then remains stable as the PEG molecular weight increases further. It was in good agreement with the conductivity trend as aforementioned. Higher conductivity indicates a faster electron movement, thereby meaning a higher regeneration rate of the oxidized dye [105]. The J_sc_ increased as the regeneration rate of the oxidized dye increased. The difference in the FF of all of the DSSCs was insignificant. The DSSC based on the PU−PEG 2000 showed the highest efficiency, followed by the DSSC based on the PU−PEG 4000, PU−PEG 6000, PU−PEG 8000, and lastly, the PU−PEG 10,000.

### 3.10. Lifespan of DSSCs

**Figure 10:**
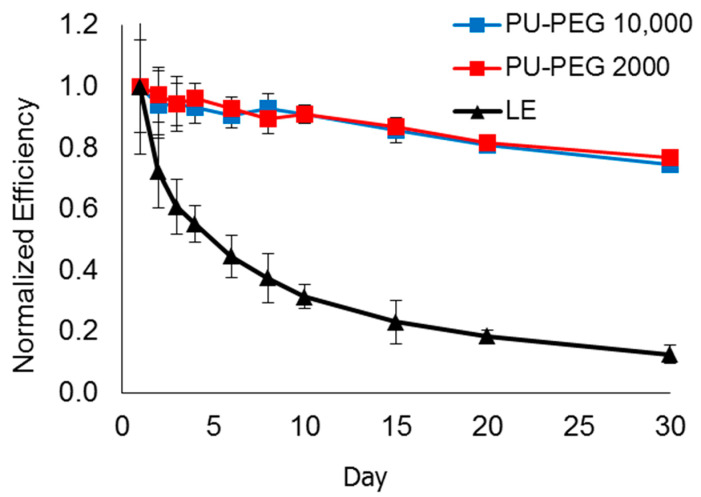
Lifespan of DSSCs based on PU matrices with different PEG molecular weights for 30 days.

Figure 10 shows the lifespan of DSSCs based on the liquid electrolyte (LE), PU−PEG 10,000 and PU−PEG 2000: normalized efficiency vs. day. Normalization changes the values of efficiencies to a common scale without distorting differences in the ranges of values. This allows an improved qualitative and quantitative comparison in graphical displays. The results indicated that the lifespan of the DSSCs based on the QSE (PU−PEG 10,000 and PU−PEG 2000) was improved tremendously if compared to the DSSC based on the liquid electrolyte (the composition of the LE was similar to the LE absorbed by PU−PEG 10,000 and PU−PEG 2000). The results also indicated that the lifespan of the DSSC with PU−PEG 10,000 was similar to that with PU−PEG 2000.

## 4. Conclusions

Reducing the PEG molecular weight improved the efficiency of the DSSC. The effect of the PEG molecular weight on the lifespan and stability of the DSSC was insignificant. However, it was significantly improved as compared to the DSSC based on a liquid electrolyte. The improvement in the efficiency was mainly due to the improvement in the J_sc_. Reducing the PEG molecular weight led to an increase in the polymer matrix absorbency and decrease in crystallinity, which contributed to the improvement in the conductivity and ion diffusion coefficient.

## Figures and Tables

**Table 1:** Formulation of PU matrices of different PEG molecular weight.

Label	Molecular Weight, gmol^−1^	PEG, g	MDI, g	PC, g	S−PANi, g	NCO:OH
PU−PEG 10,000	10,000	1	0.3	1	0.15	12.0
PU−PEG 8000	8000	1	0.3	1	0.15	9.6
PU−PEG 6000	6000	1	0.3	1	0.15	7.3
PU−PEG 4000	4000	1	0.3	1	0.15	4.8
PU−PEG 2000	2000	1	0.3	1	0.15	2.4

**Table 2:** Photovoltaic performance of DSSCs based on PU matrices with different PEG molecular weights.

	V_oc_, mV	J_sc_, mAcm^−2^	FF	Ƞ (%)
PU−PEG 2000	720 ± 04	4.52 ± 0.11	0.63 ± 0.02	3.41 ± 0.15
PU−PEG 4000	721 ± 06	4.41 ± 0.16	0.63 ± 0.01	3.33 ± 0.18
PU−PEG 6000	715 ± 12	4.24 ± 0.09	0.63 ± 0.01	3.18 ± 0.16
PU−PEG 8000	716 ± 05	4.13 ± 0.06	0.63 ± 0.01	3.11 ± 0.10
PU−PEG 10,000	708 ± 10	4.13 ± 0.14	0.65 ± 0.02	3.17 ± 0.16

## Data Availability

Not applicable.

## References

[1] Mathew S., Yella A., Gao P., Humphry-Baker R., Curchod B.F.E., Ashari-Astani N., Tavernelli I., Rothlisberger U., Nazeeruddin M.K., Grätzel M. (2014). Dye-sensitized solar cells with 13% efficiency achieved through the molecular engineering of porphyrin sensitizers. Nat. Chem..

[2] Huo Z., Dai S., Wang K., Kong F., Zhang C., Pan X., Fang X. (2007). Nanocomposite gel electrolyte with large enhanced charge transport properties of an I3-/I-redox couple for quasi-solid-state dye-sensitized solar cells. Sol. Energy Mater. Sol. Cells.

[3] Kamenan K.A., Jagadeesh A., Kre N.R., Assanvo E.F., Soman S., Unni K.N.N. (2021). Natural rubber (Hevea Brasiliensis)-based quasi-solid electrolyte as a potential candidate for arresting recombination and improving performance in aqueous dye-sensitized solar cells. J. Mater. Sci. Mater. Electron..

[4] Raut P., Kishnani V., Mondal K., Gupta A., Jana S.C. (2022). A review on gel polymer electrolytes for dye-sensitized solar cells. Micromachines.

[5] Jena A., Mohanty S.P., Kumar P., Naduvath J., Lekha P., Das J., Narula H.K., Mallick S., Bhargava P., Jena A. (2012). Dye sensitized solar cells: A review. Trans. Indian Ceram. Soc..

[6] Wu J., Lan Z., Hao S., Li P., Lin J. (2008). Progress on the electrolytes for dye-sensitized solar cells. Pure Appl. Chem..

[7] Wu J., Lan Z., Lin J., Huang M., Hao S., Fang L. (2007). Influence of solvent on the poly (acrylic acid)-oligo-(ethylene glycol) polymer gel electrolyte and the performance of quasi-solid-state dye-sensitized solar cells. Electrochim. Acta.

[8] Zheng J. (2017). Graphene tailored polymer gel electrolytes for 9.1% efficiency quasi-solid-state dye-sensitized solar cells. J. Power Sources.

[9] Wu B.J., Lan Z., Lin J., Huang M., Hao S., Sato T., Yin S. (2007). A Novel Thermosetting gel electrolyte for stable quasi-solid-state dye-sensitized solar cells. Adv. Mater..

[10] Li Q., Li H., Jin X., Chen Z. (2018). PEDOT and derivatives tailored conducting gel electrolytes for high-efficiency quasi-solid-state dye-sensitized solar cells. Electrochim. Acta.

[11] Li P., Yuan S., Tang Q., He B. (2014). Robust conducting gel electrolytes for efficient quasi-solid-state dye-sensitized solar cells. Electrochim. Acta.

[12] Lan Z., Wu J., Lin J., Huang M., Yin S., Sato T. (2007). Influence of molecular weight of PEG on the property of polymer gel electrolyte and performance of quasi-solid-state dye-sensitized solar cells. Electrochim. Acta.

[13] Yuan S., Tang Q., He B., Yu L. (2015). Conducting gel electrolytes with microporous structures for efficient quasi-solid-state dye-sensitized solar cells. J. Power Sources.

[14] Tang Z., Liu Q., Tang Q., Wu J., Wang J., Chen S., Cheng C., Yu H., Lan Z., Lin J. (2011). Electrochimica acta preparation of PAA-G-CTAB/PANI polymer based gel-electrolyte and the application in quasi-solid-state dye-sensitized solar cells. Electrochim. Acta.

[15] Li Q., Tang Q., He B., Yang P. (2014). Full-ionic liquid gel electrolytes: Enhanced photovoltaic performances in dye-sensitized solar cells. J. Power Sources.

[16] Yang Y., Tao J., Jin X., Qin Q. (2011). New microporous polymer electrolyte based on polysiloxane grafted with imidazolium iodide moieties for DSSC. Int. J. Photoenergy.

[17] Yang H., Ileperuma O.A., Shimomura M., Murakami K. (2009). Effect of ultra-thin polymer membrane electrolytes on dye-sensitized solar cells. Sol. Energy Mater. Sol. Cells.

[18] Priya A.R.S., Subramania A., Jung Y., Kim K. (2008). High-performance quasi-solid-state dye-sensitized solar cell based on an electrospun PVdF-HFP membrane electrolyte. Langmuir.

[19] Angaiah S., Murugadoss V., Arunachalam S., Panneerselvam P., Krishnan S. (2018). Influence of various ionic liquids embedded electrospun polymer membrane electrolytes on the photovoltaic performance of DSSC. Eng. Sci..

[20] Vinoth S., Kanimozhi G., Kumar H., Srinadhu E.S., Satyanarayana N. (2019). High conducting nanocomposite electrospun PVDF-HFP/TiO 2 quasi-solid electrolyte for dye-sensitized solar cell. J. Mater. Sci. Mater. Electron..

[21] Zhao J., Jo S., Kim D. (2014). Photovoltaic performance of dye-sensitized solar cells assembled with electrospun polyacrylonitrile/silica-based fibrous composite membrance. Electrochim. Acta.

[22] Murugadoss V., Arunachalam S., Elayappan V., Angaiah S. (2018). Development of electrospun PAN/CoS nanocomposite membrane electrolyte for high-performance DSSC. Ionics.

[23] Dissanayake S.S., Dissanayake M.A.K.L., Seneviratne V.A. (2016). Performance of dye sensitized solar cells fabricated with electrospun polymer nanofiber based electrolyte. Mater. Today Proc..

[24] Saidi N.M., Goh Z.L., Arif H.M., Farhana N.K., Ramesh S., Ramesh K. (2021). Consolidation of ion promoters into quasi solid-state (QSS) polymer electrolytes for dye-sensitized solar cells (DSSCs). Solid State Ion..

[25] Chen K., Liu C., Huang H., Tsai C., Chen F. (2013). Polyvinyl butyral-based thin film polymeric electrolyte for dye-sensitized solar cell with long-term stability. Int. J. Electrochem. Sci..

[26] Li Q., Chen X., Tang Q., Cai H., Qin Y., He B., Li M., Jin S., Liu Z. (2014). Enhanced photovoltaic performances of quasi-solid-state dye-sensitized solar cells using a novel conducting gel electrolyte. J. Power Sources.

[27] Weerasinghe A.M.J.S., Dissanayake M.A.K.L., Senadeera G.K.R. (2017). Application of electrospun cellulose acetate nanofibre membrane based quasi-solid state electrolyte for dye sensitized solar Cells. Ceylon J. Sci..

[28] Sj L., Pw L., Fy H., Kw C., Yl T. (2015). Correlation between dye-sensitized solar cell performance and internal resistance using electrochemical impedance spectroscopy. Phys. Chem. Biophys..

[29] Liow K.S., Sipaut C.S., Jafarzadeh M. (2018). Polypyrrole- and polyaniline-surface modified nanosilica as quasi-solid state electrolyte ingredients for dye-sensitized solar cells. J. Mater. Sci. Mater. Electron..

[30] Liow K.S., Sipaut C.S., Mansa R.F., Ung M.C., Jafarzadeh M. (2018). Formulated quasi-solid state electrolyte based on polypyrrole/polyaniline—Polyurethane nanocomposite for dye-sensitized solar cell. J. Mater. Sci. Mater. Electron..

[31] Boonsin R., Sudchanham J., Panusophon N., Sae-heng P. (2012). Dye-sensitized solar cell with poly (acrylic acid-co-acrylonitrile)-based gel polymer electrolyte. Mater. Chem. Phys..

[32] Sethupathy M., Pandey P., Manisankar P. (2014). Development of quasi-solid-state dye-sensitized solar cell based on an electrospun polyvinylidene fluoride—Polyacrylonitrile membrane electrolyte. J. Appl. Polym. Sci..

[33] Vinoth S., Kanimozhi G., Hari Prasad K., Kumar H., Srinadhu E.S., Satyanarayana N. (2018). Enhanced ionic conductivity of electrospun nanocomposite (PVDF-HFP 1 TiO_2_ nanofibers fillers) polymer fibrous membrane electrolyte for DSSC application. Polym. Compos..

[34] Spasova M., Manolova N., Markova N., Rashkov I. (2016). Superhydrophobic PVDF and PVDF-HFP nanofibrous mats with antibacterial and anti-biofouling properties. Appl. Surf. Sci..

[35] He S., Zhai S., Zhang C., Xue Y., Yang W., Lin J. (2019). Effect of sulfonation degree and PVDF content on the structure and transport Properties of SPEEK/PVDF blend membranes. Polymers.

[36] Bliznyuk V.N., Baig A., Singamaneni S., Pud A.A., Fatyeyeva K.Y., Shapoval G.S. (2005). Effects of surface and volume modification of poly (vinylidene fluoride) by polyaniline on the structure and electrical properties of their composites. Polymer.

[37] Wang X., Xiao C., Liu H., Huang Q., Hao J., Fu H. (2018). Poly (vinylidene fluoride-hexafluoropropylene) porous membrane with controllable structure and applications in efficient oil/water separation. Materials.

[38] Lee J., Choi H., Park S., Won D., Park H., Kim J., Lee C. (2010). Fabrications of poly (vinylidenefluoride-co-hexafluoropropylene) nanofibers containing inorganic filler by electrospinning technique and its application to dye-sensitized solar cells. Mol. Cryst. Liq. Cryst..

[39] Ibrahim S., Ahmad A., Mohamed N.S. (2018). Comprehensive studies on polymer electrolyte and dye-sensitized solar cell developed using castor oil-based polyurethane. J. Solid State Electrochem..

[40] Su’ait M.S., Jumaah F.N., Faizzi H.M., Mamat S., Ludin N.A., Farhan W.A., Haron A., Atifah N., Latif M.N., Badri K.H. (2018). Palm-based polyurethane-ionic liquid gel polymer electrolyte for quasi-solid state dye sensitized solar cell. Ind. Crops Prod..

[41] Lee H.S., Han C.H., Sung Y.M., Sekhon S.S., Kim K.J. (2011). Gel electrolyte based on UV-cured polyurethane for dye-sensitized solar cells. Curr. Appl. Phys..

[42] Chuang P.Y., Chang L.Y., Chuang C.N., Chen S.H., Lin J.J., Ho K.C., Hsieh K.H. (2016). A novel gel electrolyte based on polyurethane for highly efficient in dye-sensitized solar cells. J. Polym. Res..

[43] Li Q., Chen H., Li P., Qin Y., Li M., He B., Chu L., Tang Q. (2015). Quasi-solid-state dye-sensitized solar cell from polyaniline integrated poly (hexamethylene diisocynate tripolymer/polyethylene glycol) gel electrolyte. J. Mater. Chem. A.

[44] Rayung M., Aung M.M., Su’Ait M.S., Chuah Abdullah L., Ahmad A., Lim H.N. (2020). Performance analysis of jatropha oil-based polyurethane acrylate gel polymer electrolyte for dye-sensitized solar cells. ACS Omega.

[45] Liow K.S., Sipaut C.S., Ung M.C., Dayou J. (2020). Effect of incorporating different polyaniline-surface modified nanosilica content into polyurethane-based quasi-solid-state electrolyte for dye-sensitized solar cells. J. Appl. Polym. Sci..

[46] Wen T.C., Du Y.L., Digar M. (2002). Compositional effect on the morphology and ionic conductivity of thermoplastic polyurethane based electrolytes. Eur. Polym. J..

[47] Sa’Adun N.N., Subramaniam R., Kasi R. (2014). Development and characterization of poly(1-vinylpyrrolidone-co-vinyl acetate) copolymer based polymer electrolytes. Sci. World J..

[48] Wong C.S., Badri K.H., Ataollahi N., Law K.P., Su M.S., Hassan N.I. (2014). Synthesis of new bio-based solid polymer electrolyte effect of NCO/OH ratio on their chemical, thermal properties and ionic conductivity. Int. J. Chem. Mol. Eng..

[49] Chen C.C., Ting C.C. (2013). Photoelectrode fabrication of dye-sensitized nanosolar cells using multiple spray coating technique. Int. J. Photoenergy.

[50] Chen Y.S., Lee J.N., Tsai S.Y., Ting C.C. (2008). Manufacture of dye-sensitized nano solar cells and THEIR I-V curve measurements. Mater. Sci. Forum.

[51] Chang H., Chen W.A., Kao M.J. (2007). An investigation of TiO_2_ thin film in dye sesitized solar cells by electrophoresis method. Mater. Sci. Forum.

[52] Ung M.C., Sipaut C.S., Dayou J., Liow K.S., Kulip J., Mansa R.F. (2017). Fruit Based Dye Sensitized Solar Cells. IOP Conference Series: Materials Science and Engineering.

[53] Wang Z., Yang X., Zhou Y., Liu C. (2013). Mechanical and thermal properties of polyurethane films from peroxy-acid wheat straw lignin. BioResources.

[54] Strankowski M., Strankowska J., Gazda M., Piszczyk L., Nowaczyk G., Jurga S. (2012). Thermoplastic polyurethane/(organically modified montmorillonite) nanocomposites produced by in situ polymerization. Express Polym. Lett..

[55] Kannan M., Bhagawan S.S., Thomas S., Joseph K. (2013). Thermogravimetric analysis and differential scanning calorimetric studies on nanoclay-filled TPU/PP blends. J. Therm. Anal. Calorim..

[56] Kang S.H., Ku D.C., Lim J.H., Yang Y.K., Kwak N.S., Hwang T.S. (2005). Characterization for pyrolysis of thermoplastic polyurethane by thermal analyses. Macromol. Res..

[57] Vega-baudrit J., Carballo S.M., De Adhesión L., De Alicante U., Rica C., El-Sonbati A. (2012). Thermoplastic polyurethanes-fumed silica composites: Influence of NCO/OH in the study of thermal and rheological properties and morphological characteristics. Thermoplastic—Composite Materials.

[58] Agarwala S., Thummalakunta L.N.S.A., Cook C.A., Peh C.K.N., Wong A.S.W., Ke L., Ho G.W. (2011). Co-existence of LiI and KI in filler-free, quasi-solid-state electrolyte for efficient and stable dye-sensitized solar cell. J. Power Sources.

[59] Jawad M., Al-Ajaj E., Suhail M., Majid S.R. (2014). Efficiency enhancement of photovoltaic performance of quasi-solid state dye Sensitized solar cell with TPAI and KI binary iodide salt mixture. Adv. Phys. Theor. Appl..

[60] Yang Y., Hu H., Zhou C.H., Xu S., Sebo B., Zhao X.Z. (2011). Novel agarose polymer electrolyte for quasi-solid state dye-sensitized solar cell. J. Power Sources.

[61] Liang G., Zhong Z., Qu S., Wang S., Liu K., Wang J., Xu J. (2013). Novel in situ crosslinked polymer electrolyte for solid-state dye-sensitized solar cells. J. Mater. Sci..

[62] Hao F., Lin H., Zhang J., Zhuang D., Liu Y., Li J. (2010). Influence of iodine concentration on the photoelectrochemical performance of dye-sensitized solar cells containing non-volatile electrolyte. Electrochim. Acta.

[63] Hauch A., Georg A. (2001). Diffusion in the electrolyte and charge-transfer reaction at the platinum electrode in dye-sensitized solar cells. Electrochim. Acta.

[64] Hsu H.L., Tien C.F., Yang Y.T., Leu J. (2013). Dye-sensitized solar cells based on agarose gel electrolytes using allylimidazolium Iodides and environmentally benign solvents. Electrochim. Acta.

[65] Krawczak E., Zdyb A. (2019). The influence of the dye adsorption time on the DSSC performance. E3S Web Conf..

[66] Yu H., Zhang S., Zhao H., Will G., Liu P. (2009). An efficient and low-cost TiO2 compact layer for performance improvement of dye-sensitized solar cells. Electrochim. Acta.

[67] Huang K.C., Vittal R., Ho K.C. (2010). Effects of crown ethers in nanocomposite silica-gel electrolytes on the performance of quasi-solid-state dye-sensitized solar cells. Sol. Energy Mater. Sol. Cells.

[68] Park J.H., Jun Y., Yun H.G., Lee S.Y., Kang M.G. (2008). Fabrication of an efficient dye-sensitized solar cell with stainless steel substrate. J. Electrochem. Soc..

[69] Malay O., Oguz O., Kosak C., Yilgor E., Yilgor I. (2013). Polyurethaneurea E silica nanocomposites: Preparation and investigation of the structure E property behavior. Polymer.

[70] Petrini P., Fare S., Piva A., Tanzi M. (2003). Design, synthesis and properties of polyurethane hydrogels for tissue engineering. J. Mater. Sci. Mater. Med..

[71] Ren Z.Y., Wu H.P., Ma J.M., Ma D.Z. (2004). FTIR studies on the model polyurethane hard segments based on a new waterborne chain extender dimethylol butanoic acid. Chin. J. Polym. Sci..

[72] Wong C.S. (2012). Chemical analyses of palm kernel oil-based polyurethane prepolymer. Mater. Sci. Appl..

[73] Asefnejad A., Khorasani M.T., Behnamghader A., Farsadzadeh B., Bonakdar S. (2011). Manufacturing of biodegradable polyurethane scaffolds based on polycaprolactone using a phase separation method: Physical properties and in vitro assay. Int. J. Nanomed..

[74] Shriner R.L., Hermann C.K.F., Morrill T.C., Curtin D.Y., Fuson R.C. (2004). The Systematic Identification of Organic Compounds.

[75] Gogoi R., Alam S. (2013). Study of effect of NCO/OH molar ratio and molecular weight of polyol on the physico-mechanical properties of polyurethane plaster cast study of effect of NCO/OH molar ratio and molecular weight of polyol on the physico-mechanical properties of polyure. World Appl. Sci. J..

[76] Gite V.V., Mahulikar P.P., Hundiwale D.G., Kapadi U.R. (2004). Polyurethane coatings using trimer of isophorone diisocyanate. J. Sci. Ind. Res..

[77] Plisko T.V., Bildyukevich A.V., Usosky V.V., Volkov V.V. (2016). Influence of the concentration and molecular weight of polyethylene glycol on the structure and permeability of polysulfone hollow fiber membranes. Pet. Chem..

[78] Yan W., Han Z.J., Phung B.T., Faupel F., Ostrikov K.K. (2014). High-voltage insulation organic-inorganic nanocomposites by plasma polymerization. Materials..

[79] Xia H.S., Song M., Zhang Z.Y., Richardson M. (2007). Microphase separation, stress relaxation, and creep behavior of polyurethane nanocomposites. J. Appl. Polym. Sci..

[80] Deepa P., Jayakannan M. (2006). Microporous polyurethane: Synthesis and investigation of the mechanism of the pore formation. J. Polym. Sci. Part B Polym. Phys..

[81] Chen K., Leon Yu T., Chen Y., Lin T., Liu W. (2001). Soft- and hard-segmental phase segregation of polyester-based polyurethane. J. Polym. Res..

[82] Silver J.H., Myers C.W., Lim F., Cooper S.L. (1994). Effect of polyol molecular-weight on the physical-properties and hemocompatibility of polyurethanes containing polyethylene oxide macroglycols. Biomaterials.

[83] Lin Y., Chou N., Chen K., Ho G., Chang C., Wang S., Chu S., Hsieh K. (2008). Effect of soft segment lenght on properties of hydrophilic/hydrophobic polyurethanes. Polym. Int..

[84] Trovati G., Sanches E.A., Neto S.C., Mascarenhas Y.P., Chierice G.O. (2010). Characterization of polyurethane resins by FTIR, TGA, and XRD. J. Appl. Polym. Sci..

[85] Amado F.D.R., Rodrigues L.F., Rodrigues M.A.S., Bernardes A.M., Ferreira J.Z., Ferreira C.A. (2005). Development of polyurethane/polyaniline membranes for zinc recovery through electrodialysis. Desalination.

[86] Wen T.C., Hung S.L., Digar M. (2001). Effect of polypyrrole on the morphology and ionic conductivity of TPU electrolyte containing LiClO4. Synth. Met..

[87] Bagdi K., Molnár K., Sajó I., Pukánszky B. (2011). Specific interactions, structure and properties in segmented polyurethane elastomers. Express Polym. Lett..

[88] Çakmak E.G., Dalgakiran D., Güner F.S. (2018). Castor oil and PEG-based shape memory polyurethane films: Effect of chain extender amount on some polymer properties and performance. Turkish J. Chem..

[89] Pielichowski K., Flejtuch K. (2002). Differential scanning calorimetry studies on poly (ethylene glycol) with different molecular weights for thermal energy storage materials. Polym. Adv. Technol..

[90] Majumdar R., Alexander K.S., Riga A.T. (2009). Physical characterization of polyethylene glycols by thermal analytical technique and the effect of humidity and molecular weight. Pharmazie.

[91] Rodrigues P.C., Akcelrud L. (2003). Networks and blends of polyaniline and polyurethane: Correlations between composition and thermal, dynamic mechanical and electrical properties. Polymer.

[92] Gurunathan T., Rao C.R.K., Narayan R., Raju K.V.S.N. (2013). Polyurethane conductive blends and composites: Synthesis and applications perspective. J. Mater. Sci..

[93] Oka O., Kiyohara O. (1993). Electrical and mechanical properties of crosslinked polyanilines. Synth. Met..

[94] Amado F.D.R., Rodrigues L.F., Forte M.M., Ferreira C. (2006). Properties evaluation of the membranes synthesized with castor oil polyurethane and polyaniline. Polym. Eng. Sci..

[95] Das S., Pandey P., Mohanty S., Nayak S.K. (2015). Materials Express. Mater. Express.

[96] Semsarzadeh M.A., Navarchian A.H. (2003). Effects of NCO/OH ratio and catalyst concentration on structure, thermal stability, and crosslink density of poly (urethane-isocyanurate). J. Appl. Polym. Sci..

[97] Li X., Zhang D., Yin X.J., Chen S., Shi J., Sun Z., Huang S. (2011). The effects of polymer gel electrolyte composition on performance of quasi-solid-state dye-sensitized solar cells. J. Solid State Electrochem..

[98] Yoshikawa H., Hino T., Kuramoto N. (2006). Effect of temperature and moisture on electrical conductivity in polyaniline/polyurethane (PANI/PU) blends. Synth. Met..

[99] Weber L.M., Lopez C.G., Anseth K.S. (2009). Effects of PEG hydrogel crosslinking density on protein diffusion and encapsulated islet survival and function. J. Biomed. Mater. Res. Part A.

[100] Ye Y.S., Chen W.Y., Huang Y.J., Cheng M.Y., Yen Y.C., Cheng C.C., Chang F.C. (2010). Preparation and characterization of high-durability zwitterionic crosslinked proton exchange membranes. J. Memb. Sci..

[101] Gopakumar S., Gopinathan Nair M.R. (2005). Swelling characteristics of NR/PU Block copolymers and the effect of NCO/OH ratio on swelling behaviour. Polymer.

[102] Suriwong T., Thongtem T., Thongtem S. (2015). CuAlO_2_ powder dispersed in composite gel electrolyte for application in quasi-solid state dye-sensitized solar cells. Mater. Sci. Semicond. Process..

[103] Hou R., Yuan S., Ren X., Zhao Y., Wang Z., Zhang M., Li D., Shi L. (2015). Effects of acetyl acetone-typed co-adsorbents on the interface charge recombination in dye-sensitized solar cell photoanodes. Electrochim. Acta.

[104] Zhang H., Fan J., Iqbal Z., Kuang D.B., Wang L., Cao D., Meier H. (2013). Anti-recombination organic dyes containing dendritic triphenylamine moieties for high open-circuit voltage of DSSCs. Dyes Pigment..

[105] Xiang W., Fang Y., Lin Y., Fang S. (2011). Polymer-metal complex as gel electrolyte for quasi-solid-state dye-sensitized solar cells. Electrochim. Acta.

